# College and Hospital Notes

**Published:** 1904-09

**Authors:** 


### COLLEGE AND HOSPITAL NOTES.

The Mercy Hospital, situated on Tifft street, Buffalo, was opened for the reception of patients the latter part of August. This building was formerly the home of William J. Connors and several changes have been made to make it conform to the needs of such an institution. About fifty patients can be accommodated and additional buildings will be put up as circumstances may require. The location is one of the best for hospital purposes, having ample grounds in an open region away from the crowded streets and avenues of the city.

On the first floor of the building are four wards, opening off the main hall, and opposite are the office, pharmacy, three private rooms and an operating room. A diet kitchen and lavatory complete the hospital suite of this floor.

Ten private rooms, with diet kitchen, dumb-waiter and lavatory, are on the second floor, and on the third are the sisters' apartments and the chapel. The two first floors open on to wide verandas, making a pleasant resting place for convalescent patients.

In the rear of the hospital a building is to be erected which will contain a dining-room and kitchen, so that no odor of cooking will reach the patients. The building will be lighted both by electricity and gas, and will be heated by steam. Many of the private rooms and all the wards have open grates, which will be pleasant on early autumn evenings, and will at the same time serve as excellent ventilators.

The hospital grounds have a frontage of 200 feet, and although the building will be of sufficient size to begin the hospital work of the Sisters of Mercy, they intend to enlarge it by additional buildings as already stated. The entire cost of ground, building and repairs has amounted to about $14,000. Excepting the hospital at the Steel Plant, conducted by the company, there is none nearer than the Emergency.

---

The medical department of the University of Buffalo will begin its fifty-ninth annual session Monday, September 26, 1904, to continue thirty weeks. The advance registration indicates a large attendance and the college was never in better condition to render adequate instruction than at the present time.

Some changes in the governing faculty have taken place since the last announcement. Professor John Parmenter has resigned the chair of anatomy and the secretaryship of the faculty, retaining, however, the professorship of clinical surgery, his name being placed at the head of the associate faculty. Professor Herbert U. Williams has been promoted from the associate to the governing faculty and retains the title of professor of pathology and bacteriology and curator of the museum. Professor Herbert M. Hill has been appointed secretary of the faculty, at the same time retaining his chair as professor of chemistry, toxicology and physics. The college is prepared to give a four years' graded course with superb laboratory and clinical instruction.

---

Dr. John Punton, superintendent of the Punton Sanitarium, located at Kansas City, Mo., announces that in response to an increased demand for accommodations by patients he is building a large addition to the institution over which he presides, and otherwise improving it with a view to making it one of the best of its kind in the country.

